# Induction of Inflammation by *West Nile virus* Capsid through the Caspase-9 Apoptotic Pathway

**DOI:** 10.3201/eid0812.020224

**Published:** 2002-12

**Authors:** Joo-Sung Yang, Mathura P. Ramanathan, Karuppiah Muthumani, Andrew Y. Choo, Sung-Ha Jin, Qian-Chun Yu, Daniel S. Hwang, Daniel K. Choo, Mark D. Lee, Kesen Dang, WaixingTang, J. Joseph Kim, David B. Weiner

**Affiliations:** * University of Pennsylvania, Philadelphia, Pennsylvania, USA; † Viral Genomix, Inc., Philadelphia, Pennsylvania, USA

**Keywords:** West Nile Virus, Flavivirus, capsid protein, apoptosis, pathogenesis

## Abstract

*West Nile virus* (WNV) is a member of the *Flaviviridae* family of vector-borne pathogens. Clinical signs of WNV infection include neurologic symptoms, limb weakness, and encephalitis, which can result in paralysis or death. We report that the WNV-capsid (Cp) by itself induces rapid nuclear condensation and cell death in tissue culture. Apoptosis is induced through the mitochondrial pathway resulting in caspase-9 activation and downstream caspase-3 activation. Capsid gene delivery into the striatum of mouse brain or interskeletal muscle resulted in cell death and inflammation, likely through capsid-induced apoptosis in vivo. These studies demonstrate that the capsid protein of WNV may be responsible for aspects of viral pathogenesis through induction of the apoptotic cascade.

*West Nile virus* (WNV) is a member of the *Flaviviridae* family*,* which includes *St. Louis encephalitis virus*, Kunjin virus, *yellow fever virus*, *Dengue virus*, and *Japanese encephalitis virus* (1). WNV, a single-stranded RNA virus, was initially isolated in the West Nile region of Uganda in 1937 (1) and has become prevalent in Africa, Asia, and Europe. Since its introduction into the United States in summer 1999, the sudden and rapid spread of this virus in the United States has caused much concern. WNV has been reported in infected mothers’ breast milk, and WNV transmission by organ transplantation and transfusion has been documented. Clearly, WNV infection is not only a regional public health problem, but a global health issue (2). However, we lack a clear understanding of WNV pathogenesis, and little specific treatment exists for WNV infection. Therefore, a clearer understanding of WNV is necessary in order to identify new strategies to treat or prevent this viral infection (3).

Here we report on an unexpected role for WNV capsid (Cp) in viral-induced pathogenesis. We observed that the WNV-Cp protein is a pathogenic protein, which drives apoptosis in vitro through the mitochodrial/caspase-9 pathway. We also observed that expression of Cp protein in mouse muscle resulted in apoptosis and inflammation of muscle cells. More importantly, direct in vivo expression of WNV-Cp protein in mouse brain resulted in an induction of apoptosis similar to what is observed in natural infection. These results provide evidence of a link between WNV-Cp protein and WNV pathogenesis in vivo.

## Materials and Methods

### Cloning and Expression Analysis of WNV-Cp Gene

The cloning of a synthetic WNV-Cp gene based on the reported NY-99 infectious strain was described earlier (4). Western blot analysis was performed as previously described (4). For a caspase-9–specific test, 5 μg of pcWNV-Cp-DJY or pcWNV-CpWT was cotransfected with a dominant negative caspase-9 (DN caspase-9) construct, and cleavage of procaspase-9 protein was determined by Western blot analysis with antihuman caspase-9 antibody (MBL, Nagoya, Japan). DN caspase-9 (provided courtesy of Emad S. Alnmeri, Thomas Jefferson University, Philadelphia, PA) has been reported to inhibit the caspase cascade (5). The localization pattern of capsid expression was analyzed by immunofluorescent assay in HeLa, 293-T, RD, or SH-SY5Y cells by using anti-His tag antibody as described (6).

### Observations with Electron Microscope

RD cells transfected with pcWNV-Cp-DJY or pcDNA3.1 plasmid DNA were processed for transmission electron microscope analysis as described (7,8). Semithin (1.0-μm) sections were stained with toluidine blue, and photographed with Ektachrome 160T film (Eastman Kodak Co., Rochester, NY). Ultrathin sections were stained with uranyl acetate and lead citrate, and observed with a Philips CM-100 electron microscope, operated at 60 Kv.

### TUNEL Assay and Annexin V Staining

In vitro apoptosis in individual cells was determined by terminal desoxyriboxyl-desoxyriboxyl transferase–mediated DVTP nick-end labeling (TUNEL) assay with the In Situ Cell Death Assay Kit (Roche Diagnostic Corp., Indianapolis, IN) and visualized by fluorescent microscopy. Apoptosis induction by the expression of capsid was also determined by annexin V staining procedure followed by fluorescence-activated cell sorter analysis. Cells were transfected with the WNV-Cp–enhanced green fluorescent protein (EGFP) fusion construct or pcDNA3.1. Forty-eight hours after transfection, the cells were stained with phycoerythrin-conjugated annexin V. Only EGFP-expressing cells were analyzed and the data were acquired by using the CellQuest software package (Becton-Dickinson, and Co., Franklin Lakes, NJ).

### Mouse Muscle Injection

Female 6- to 8-week-old Balb/c mice (Charles River Laboratories, Inc., Wilmington, MA) were injected in the tibialis muscle with 100 μg of pcWNV-Cp-DJY or pcDNA3.1 in phosphate-buffered saline (PBS) and 0.25% bupivicaine-HCl (Sigma-Aldrich Corp., St. Louis, MO) as described (9). After 48 h, the tibialis muscle was harvested and embedded in OCT Compound (Sakura Finetek U.S.A., Inc., Torrance, CA). Muscle sections were prepared by cryosectioning and stored at –20°C until assayed. For pathologic observation, tissue sections were stained with hematoxylin/eosin (9).

### DNA Injection into Mouse Brain

Balb/c mice were anaesthetized with ketamine/xylazine (70 mg/kg of ketamine, 7 mg/kg of xylazine). Using a Hamilton syringe (Hamilton Co., Reno, NV) with a 30-gauge removable needle, 5 μg of pcWNV-Cp–DJY or pcDNA3.1 DNA, in 5 μl of endotoxin-free water and 0.25% of bupivicaine-HCl in PBS was injected into the frontal cortex (striatum) with a small animal stereotactic apparatus (Kopf Instruments, Tujunga, CA) as described (10). The DNA was injected for 3 min; the needle was left in the place for 1 min and then withdrawn slowly over 1 min.

### Mouse Brain Tissue Immunohistochemistry by Using Horseradish Peroxidase (HRP)

Twenty four to 48 h postinjection, mice were deeply anesthetized and perfused transcardially with 0.1 M PBS (pH 7.2), then with 4% paraformaldehyde (PFA) in PBS. The brains were postfixed in 4% PFA for 18 h at 4°C and cryoprotected in 30% sucrose for 48 h at 4°C, then frozen and mounted for cryostat sectioning. Sections (25 μm) were serially cut in the coronal plane. The tissue sections were treated with anti-Histag antibody with appropriate secondary antibody with the counterstaining of hematoxylin. The slides were analyzed under a fluorescent microscope for TUNEL or gene expression.

###  Detection of Mictochondria-Based Apoptotic Pathways

Caspase-3 (Pharmingen, San Diego, CA), caspase-8 (FADD-like interleukin-1 beta-converting enzyme) and caspase-9–like Mch6 (MBL, Nagoya, Japan) activities were determined according to the manufacturer’s protocol. RD cells transfected with pcWNV-Cp–DJY or pcDNA3.1 were harvested and lysed at 48 h postinjection. The cell lysates (100 μg/100 μl protein) were incubated with specific substrate Ac-DEVD-AMC for caspase-3, IETD-*p*NA for caspase-8, or LEHD-*p*NA for caspase-9 for 1–2 h at 37°C. For the inhibition test, IETD-FMK or LEHD-FMK, inhibitors for caspase-8 or -9, respectively (MBL) were added to the reaction, along with the substrate, according to the protocol. The activity of released AMC or *p*NA was determined by spectrophotometer at 405 nm. The mitochondria transmembrane potential was measured by using a DePsipher assay kit (R&D Systems, Minneapolis, MN). The cells were observed under a fluorescent microscope, and the images were acquired and analyzed in an Image-Pro program (Media Cybernetics, Inc., Houston, TX).

## Results and Discussion

WNV is a vector-borne pathogen that induces encephalitis and death in WNV-endemic regions (11). Unfortunately, no specific therapy exists for WNV infection (3). Furthermore, the exact mechanisms of WNV-induced pathogenesis have not been elucidated. To attain a better understanding of possible mechanisms of WNV biology, we studied the role of the capsid gene in WNV pathogenesis.

### WNV-Cp Protein Induces Apoptosis in Cells In Vitro

**Figure 1:**
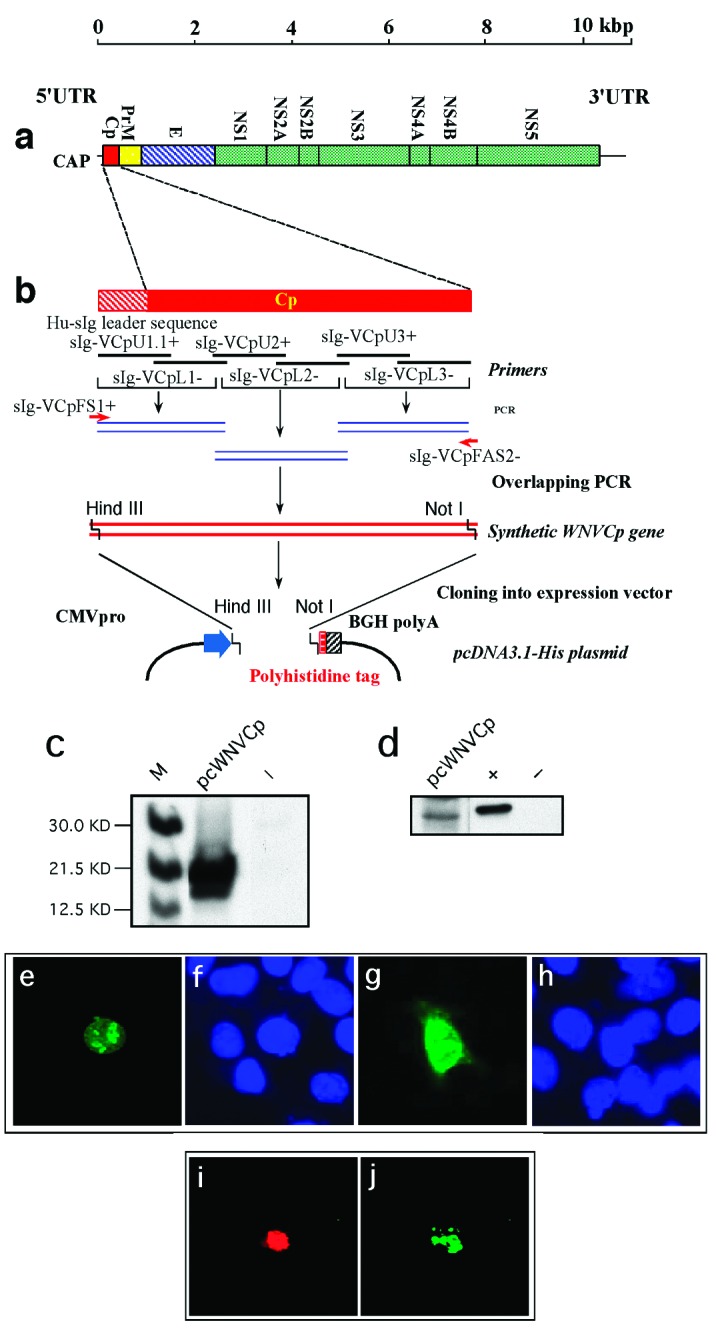
*West Nile virus* capsid (WNV-cp)-DJY protein expression induces apoptosis. Nuclear condensation was observed in HeLa cells transfected with pcWNV-Cp-DJY (a), a positive control pBax (b), or a negative control, pcDNA3.1 (c), under a 4,6-diamidino-2-phenylidole (DAPI) filter (magnification: 200X). Light microscopic observation on pcWNV-Cp-DJY (d) or pcDNA3.1 (e) plasmid transfected RD cells were examined in semithin sections stained with toluidine blue (magnification for d and e: 400X). Ultramicroscopic image of apoptotic cells were photographed from pcWNV-Cp-DJY transfected RD cells (f). DNA fragmentation in WNV capsid–expressing cell lines was examined by terminal desoxyriboxyl-desoxyriboxyl trasferase–mediated DVTP nick-end labeling (TUNEL) assay in HeLa (g), HEK 293 (i), and RD cells (k), and compared with DNA fragmentation from pcDNA3.1-transfected HeLa cells (m). Nuclear staining in HeLa (h), HEK 293 (j), and RD cells (l) were observed by using a DAPI filter and compared with control HeLa cells (n) (magnification for g through n: 400X). Cell membrane morphology changes were examined by annexin V staining/flow cytometry by using HeLa cells transfected with pcWNV-Cp-DJY or control pcDNA3.1 plasmids (o). The human neuroblastoma cell line SH-SY5Y was transfected with Bax as a positive control (p), pcWNV-Cp (r), or control plasmid (t) and examined by TUNEL assay. To visualize nuclear staining, cells transfected with pBax, pcWNV-Cp-DJY (q and s, respectively) or pcDNA3.1 (u) were stained with DAPI and observed using appropriate filters (magnification: 400X).

**Figure 2:**
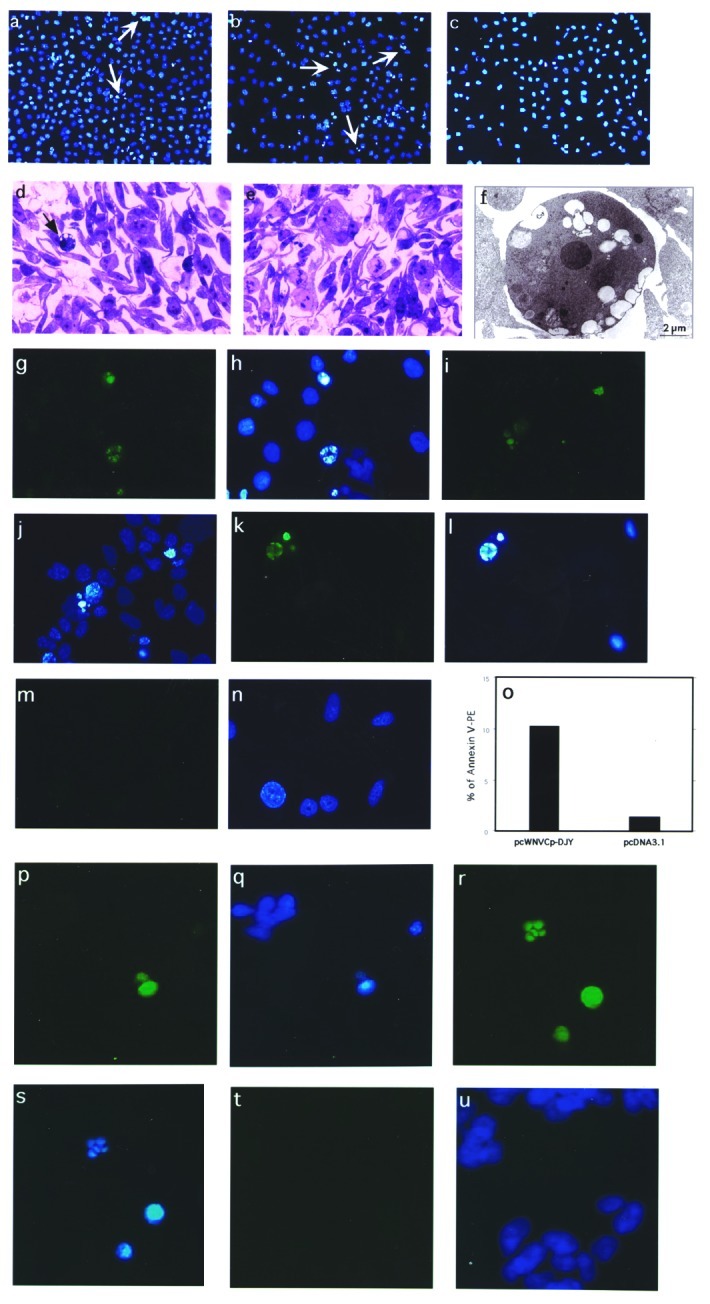
Construction and subcellular expression of *West Nile virus* (WNV)–NY1999 capsid (Cp) gene–expressing plasmid, pcWNV-Cp-DJY: a, Genomic organization of WNV-NY1999 (10,945 bp) is outlined based on the published (GenBank accession no. AF202541). b***,*** Cloning strategy for WNVCp gene–expressing plasmid, pcWNV-Cp-DJY. c***,*** In vitro translated and immunoprecipitated ^35^S-labeled WNV-Cp visualized by SDS-PAGE. WNV-Cp–specific protein synthesis was compared to control generated by the vector backbone pcDNA3.1 (-). d, Protein expression by Western blot analysis, of WNV-Cp expression in HeLa cells. Subcellular location of WNV-Cp protein, in HeLa cells transfected with pcWNV-CpWT (e,f) or pcWNV-Cp-DJY (g,h) plasmids. 16 h posttransfection, the cells were visualized by indirect immunofluorescence. Typical nuclear staining was observed with the cells expressing WNV-CpWT (e) compared to the cells expressing WNV-Cp-DJY (g). TUNEL assay on the WNV-Cp–transfected cells, indicating nuclear condensation (i) due to expression of capsid (j).

The expression of the Cp gene was examined by in vitro transcription/translation system as well as Western blot analysis. The cells expressing WNV-Cp, as well as the positive control plasmid encoding the proapoptotic protein, Bax (Figure 1b), show nuclear condensation, which is a typical feature of apoptotic cells (Figure 1c). We carried out TUNEL assays for apoptosis with a double-staining procedure. The WNV-Cp–transfected HeLa cells were simultaneously stained for TUNEL assay, which reveals nuclear condensation, and for capsid expression with rhodamine-conjugated secondary antibody. The double-positive cells (Figure 2i) (Figure 2j) indicate the induction of apoptosis specifically driven by the expression of capsid.

Cells transfected with pcWNV-Cp-DJY were further investigated by transmission electron microscopy. Examination of semithin sections (1.0 μm) stained with toluidine blue revealed typical apoptotic cells representing approximately 5% of the total cell population in pcWNV-Cp-DJY–transfected cells (Figure 1d, arrow) but not in control cells (Figure 1e). These apoptotic cells usually lost their polygonal shape as well as their contact with neighboring cells, and became round and stained exceptionally dark. Many of the apoptotic cells also exhibited clear vacuoles in the condensed cytoplasm. Fragmented, but equally condensed, apoptotic bodies were also present. Ultrastructurally, all of the apoptotic cells showed continuous plasma membranes, apparent aggregation of nuclear chromatin, highly condensed cytoplasm, and nearly intact organelles (Figure 1f). Cells transfected with pcWNV-Cp-DJY show typical features of apoptosis.

The induction of apoptosis by capsid expression was also confirmed in different human cell lines such as HeLa, 293, RD, or SH-SY5Y. All three cell lines, HeLa, 293, and RD, transfected with pcWNV-Cp-DJY were TUNEL-positive (Figure 1g, l, and k, respectively), whereas the control transfected cells were not (Figure 1m). Proapoptotic Bax expression plasmid was used as a positive control. Experiments with annexin V staining revealed that 22.9% of WNVCp-transfected cells undergo phosphatidylserine dislocalization, a typical early feature of apoptosis.

### Apoptosis by WNV-Cp In Vivo

**Figure 3:**
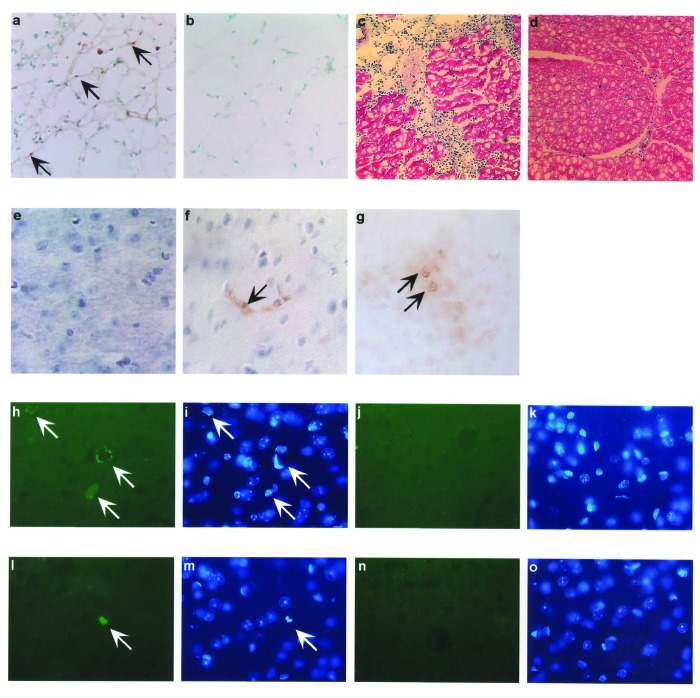
In vivo *West Nile virus* capsid (Cp) expression induces apoptosis and inflammation in mice. TUNEL assay was performed on muscle cryosections harvested from mice injected with pcWNV-Cp-DJY (a) or pcDNA3.1 (b). Hematoxylin/eosin staining was performed on mouse tibialis muscle cryosections harvested from mice injected with pcWNV-Cp-DJY (c) or pcDNA3.1 (d) at 48 h postinjection (magnification: 200X [a, b] and 40X [c,d]). Immunohistochemical analysis was performed for detection of WNVCp-DJY protein expression in mouse brain injected with pcDNA3.1 or pcWNV-Cp-DJY as detected with horseradish peroxidase (HRP) (e,f, respectively). TUNEL assay on mouse brain cryosections harvested from pcWNV-Cp-DJY injected mouse was detected with HRP (g) (magnification: 300X [e–g]). Immunohistochemical studies were performed for detection of WNV-Cp-DJY protein expression in mouse brain injected with pcWNV-Cp-DJY or pcDNA3.1 as detected by fluorescein isothiocynate stain (h, i, and j, k, respectively). TUNEL assay on mouse brain cryosections harvested from pcWNV-Cp-DJY–injected mice (l,m) or pcDNA3.1-injected mice as detected with fluorescein isothiocyanate (n,o). WNV-Cp-DJY protein expressing His-positive cells or TUNEL-positive cells were visualized under ultraviolete microscope (h or l, respectively). Nuclear staining for WNV-Cp-DJY– or pcNDA3.1-transfected cells was visualized with appropriate filters (i, m or k, o, respectively) (magnification: 630X [h through o]).

We have shown, as have other researchers, that induction of apoptosis by plasmid in vivo results in enhanced levels of proinflammatory T-cell activation (4,9,12–14). We extended these in vitro findings of the ability of WNV-Cp to induce apoptosis to an animal model in vivo by using a direct plasmid delivery method as described (9,14). At 24 h after plasmid injection, TUNEL revealed positive signals (noted by dark brown because of the HRP-DAB reaction) in pcWNV-Cp-DJY injected mouse muscle (Figure 3a, arrows) but not in control muscle (Figure 3b). We observed severe inflammation within 48 h in mouse muscle injected with pcWNV-Cp-DJY (Figure 3c) but not in the pcDNA3.1-injected mouse muscle (Figure 3d). These studies indicate that expression of Cp protein in mouse muscle resulted in apoptosis and inflammation of muscle cells in vivo. In this regard, induction of apoptosis and the resulting inflammatory cell infiltration induced by WNV-Cp expression may have an important role in viral pathogenesis.

WNV has been found in the brains (15) and cerebrospinal fluids (16) of infected patients, where it induces cell death, resulting in encephalitis (17–19). As no component of WNV had been previously implicated in in vivo cell death of neuronal tissue, we reasoned that the WNV-Cp was a possible candidate, and we sought to investigate the effects of WNV-Cp in the brain in vivo. We directly injected DNA into the brain because that approach would not be complicated by vector delivery.

Mice were injected stereotactically with WNV-Cp or control plasmid DNA and euthanized 24–48 h after injection. Sections were processed from the harvested brain samples as described in Materials and Methods. By using a monoclonal antibody specific to the His epitope contained in the plasmid vector, immunohistochemical analysis revealed that His-positive cells, as identified by HRP or fluorescein isothiocynate (FITC) were found in pcWNV-Cp-DJY–injected mouse brain (Figure 3f, h, respectively). Similar expression was absent in the brains of mice injected with control plasmid (Figure 3e, j, respectively). However, His-positive cells were detected in several areas of the cerebral cortex, including the motor cortex of the pcWNV-Cp-DJY injected mice (Figure 3f by HRP, and 3h by FITC [arrows]). Nuclear condensation, a classic feature of apoptosis, was also observed in these sections by DAPI staining (Figure 3i and 3m; arrows). As shown in Figure 3g by HRP and 3l by FITC, we observed TUNEL-positive cells in the brain sections from the pcWNV-Cp-DJY–injected mice (Figure 3g and 3l) and not in the pcDNA3.1–injected control mice (Figure 3n). These TUNEL-positive cells were localized to the specific sites of injection (Figure 3l, arrow). These results illustrate that His-expressing cells were also TUNEL-positive, showing the direct relationship of WNV-Cp expression and in vivo apoptosis. WNV-Cp protein expression and apoptosis as well as inflammation were highly reproducible in all animals studied by injection with pcWNV-Cp-DJY plasmid. These data strongly suggest that the expression of the WNV-Cp protein in the central nervous system can play a role in neuronal cell death, and this process may be important in the pathogenesis of WNV-induced encephalitis.

### Mitochondrial-Activated apoptotic Pathway

**Figure 4:**
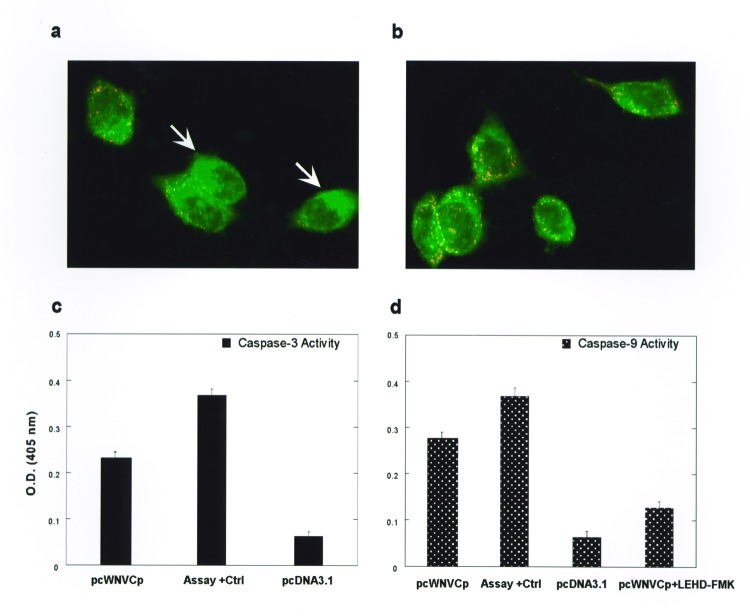
Mitochondria transmembrane potential and caspase activities measurement. HeLa-CD4 cells were transfected with pcWNV-Cp-DJY (a) or pcDNA3.1 (b), and their mitochondria transmembrane potential was measured after 48 h by ultraviolete illumination. A colorimetric caspase activity assay was performed with pcWNV-Cp-DJY– or pcDNA3.1-transfected cells for caspase-3 (c) or caspase-9 (d) activity. As a specificity control, the inhibitor LEHD-FMK for caspase-9 was added to the reactions along with relevant substrate (d). A specific positive control was used for assay validation (magnification: 1000X [a and b]).

To characterize the apoptosis pathway activated by the WNV-Cp protein, we next examined its direct effects on the disruption of the mitochondrial transmembrane potential in these cells. HeLa-CD4 cells were transfected with pcWNV-Cp-DJY or pcDNA3.1 plasmids, and the mitochondrial membrane potential was measured with a DePsipher assay kit (R&D Systems). The pcDNA3.1-transfected cells showed a normal pattern of orange-red fluorescence (Figure 4b). In contrast, green fluorescence was clearly visible in the pcWNV-Cp-DJY–transfected cells (Figure 4a, arrows).

We examined the effects of WNV-Cp on caspase-3, -8, or -9 activity. Cell lysates from pcWNV-Cp-DJY–transfected cells showed marked activity for caspase-3, illustrating substantial apoptotic induction (Figure 4c). Moreover, the cell lysate harvested from pcWNV-Cp-DJY–transfected cells was positive for caspase-9 activity, and this activity was inhibited by the addition of the caspase-9-specific inhibitor, LEHD-FMK (Figure 4d). In contrast, caspase-8 activity from these samples was not greatly increased relative to the negative control, and little effect was noted by the addition of the caspase-8–specific inhibitor, IETD-FMK (data not shown). Cell lysates from pcDNA3.1 control transfected cells show no caspase-8 (data not shown) or -9 activity (Figure 4d). These results firmly suggest that the mechanism of WNV-Cp–induced apoptosis is through the disruption of the mitochondrial transmembrane potential and the activation of caspase-9, which result ultimately in activation of the caspase-3 pathway.

### Mapping Apoptosis-Inducing Domain

**Figure 5:**
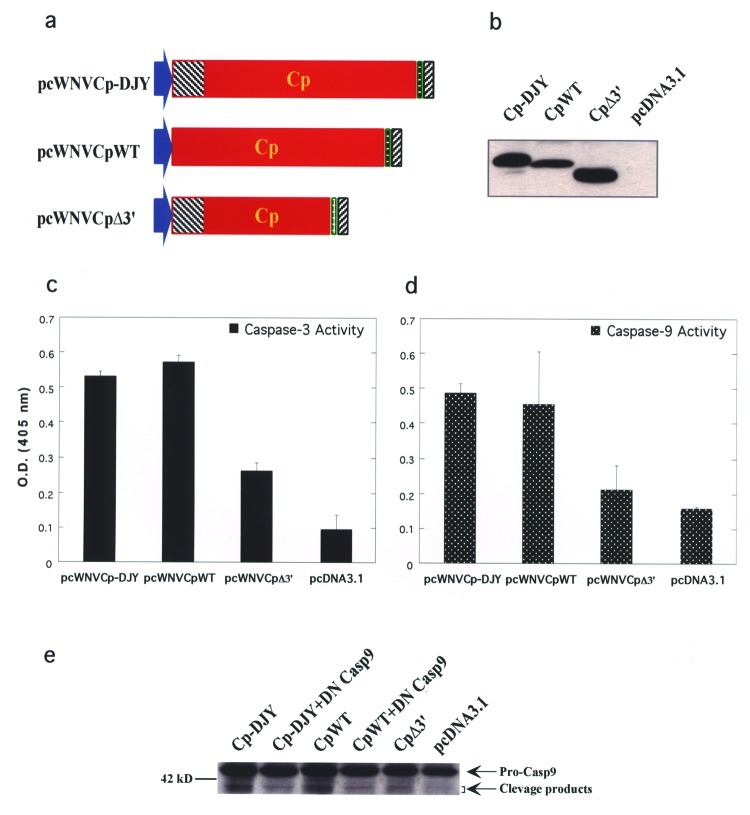
Apoptosis determining domain in WNV-Cp gene. a, Schematic diagram of pcWNV-Cp-DJY, pcWNV-CpWT, and pcWNV-Cp∆3′ constructs. b, Immunoprecipitation of in vitro translated protein from pcWNV-Cp-DJY (Cp-DJY), pcWNV-CpWT (CpWT), and pcWNV-Cp-3′ (Cp∆3′) plasmids. As a negative control, pcDNA3.1 in vitro translated supernatants were analyzed. c***,*** Colorimetric caspase-3 activity assay using pcWNV-Cp-DJY (Cp-DJY), pcWNV-CpWT (CpWT), or pcWNV-CpΔ3′ (CpΔ3’) plasmid–transfected cells. d***,*** The cell lysates were assayed for caspase-9-like activity, and the pcDNA3.1-transfected cell lysate was used as the negative control. e, Inhibition of WNV-Cp–induced apoptosis by a dominant negative (DN) caspase-9 plasmid (DN Casp9) was assayed with equal amount of cell lysates from co-transfection of pcWNV-Cp-DJY (Cp-DJY) or pcWNV-CpWT (CpWT); an expression level of pro-caspase-9 cleavage products (35–37 kD) was compared to 3′-terminal deletion mutant, pcWNV-Cp∆3′ (Cp∆3′) and pcDNA3.1 by Western blot analysis with anti-human caspase-9 mAb.

 To map the apoptosis-inducing domain, a 3′-terminal deletion mutant with deletion of 3′-terminal 55 amino acids was generated, and its integrity was tested by in vitro translation/transcription (Figure 5a,b). Furthermore, to examine whether the specific 3′-terminal region is a determinant for the observed apoptosis, plasmids were transfected into RD cells, and cell lysates were analyzed for caspase-3, -8, and -9 activities. The native WNV-Cp constructs showed strong caspase-3 activity (Figure 5c). In addition, this 3′-deletion mutant showed similarly lower induction of caspase-9 activity (Figure 5d). These data support the hypothesis that this 3′ domain plays an important role in the induction of apoptosis by the WNV-Cp protein. Furthermore, to confirm that this apoptosis-induction pathway is through caspase-9, a domant negative (DN) caspase-9 construct, which has been reported to inhibit the caspase cascade, was cotransfected wiwth pcWNV-Cp-DJY or pcWNV-CpWT and the expression level of pro-caspase-9 cleavage products (35–37 kD) was compared to the activity of the 3′-deletion mutant, pcWNV-CpΔ3′ and pcDNA3.1 in Western blot analysis. The DN caspase-9 specifically blocked the cleavage of pro-caspase-9 in pcWNV-Cp-DJY and pcWNV-CpWT cotransfected cell lysates compared to those of pcWNV-Cp-DJY or pcWNV-CpWT in transfected cell lysates (Figure 5e). Moreover, the cell lysate transfected with the 3′-terminal deletion mutant had lower cleavage of pro-caspase-9, which is related to the lower induction of caspase-3 and –9 activity as determined by protease activity assay (Figure 5c,d). Cell lysates from pcDNA3.1 control transfected cells show much less pro-caspase-9 cleavage products.

Although the apoptotic effects of wild-type WNV as well as other flaviviruses have been previously reported, the gene or genes responsible for this effect in WNV have not been described. The Cp-induced apoptosis in the brain implies that the expression of the WNV-Cp protein in the central nervous system may play an important role in initiating neuronal cell death through apoptosis-induced inflammation. Therefore, this process may be important in the pathogenesis of WNV-induced encephalitis.

In this study, the WNV-Cp protein–induced apoptosis through the destabilization of the mitochondrial transmembrane, resulting in the likely release of cytochrome c (20,21). The complex of cytochrome c/Apaf-1 recruits and activates procaspase-9 (22), not procaspase-8. Paradoxically, the karyophilicity of WNV-Cp protein does not fully explain the destabilization of the mitochondrial membrane and its ability to drive the caspase-9 apoptotic pathway. Therefore, it is possible that WNV-Cp changes the host cell transcriptional machinery, resulting in an over expression of certain proteins related to an apoptotic program, which consequently feed back to the mitochondria, or that as WNV-Cp moves from the cytoplasm to the nucleus, it may sequester or inactivate an important member of the antiapoptotic pathway or the cell cycle pathway, and thus induce the apoptotic cascade. Furthermore, the data suggest that WNV-Cp may interact with host cell proteins to induce apoptosis in the host cell. Identifying these proteins will likely give more insight into the biology of WNV. This biology likely involves the WNV-Cp 3′ region. Moreover, this flavivirus contains a capsid protein, which localizes to the nucleus. Flavivirus replication is normally cytoplasmic, although some evidence supports nuclear function as part of the viral life cycle. In fact, Kunjin virus capsid has been found in the nucleus (23). Our results identify the nuclear localizing property of the protein as a potential pathogenic attribute. Hence, the pathogenic region of the protein is localized within the 3′-terminal region. Therefore, creating a WNV isolate that no longer localizes the capsid to the nucleus may result in a virus that loses pathogenesis, providing a novel approach for vaccine studies. These results also imply that inhibiting the C-terminal region’s ability to interact with its putative ligand could be an important target for the development of new treatments for WNV infection.
